# Stage-dependent shifts in non-structural carbohydrates and C, N stoichiometry in *Setaria viridis* along a salinity gradient in the Xiaokai River irrigation area of the Yellow River Delta, China

**DOI:** 10.3389/fpls.2026.1880438

**Published:** 2026-07-15

**Authors:** Xiaozhu Wang, Xuejie Zhang, Shoujin Fan, Peipei Jiang

**Affiliations:** Shandong Provincial Key Laboratory of Plant Stress Biology and Genetic Improvement, College of Life Sciences, Shandong Normal University, Ji’nan, China

**Keywords:** ecological stoichiometry, growth stage, non-structural carbohydrates, soil properties, soil salinity gradient

## Abstract

**Introduction:**

Non-structural carbohydrates (NSC), carbon (C), nitrogen (N) and their ratio are fundamental to plant carbon metabolism and nutrient balance, yet the dynamic relationships among these indicators under salt stress remain poorly understood.

**Methods:**

We measured plant NSC content, and C, N stoichiometry in aboveground and belowground parts of *Setaria viridis*, along with soil pH, electrical conductivity, C and N content, across a salinity gradient spanning 91.5 km in the Xiaokai River irrigation area of the Yellow River Delta, China.

**Results and discussion:**

We found that plant NSC content was consistently higher in aboveground parts than in belowground parts across growth stages, indicating an aboveground−prioritized carbon strategy. For plant NSC components, soluble sugars acted as the primary drivers of NSC accumulation and dominated carbon partitioning and utilization, whereas starch played a more prominent role in carbon storage during the late growth stage than in the early growth stage, indicating a stage−dependent shift from immediate energy supply to reserve maintenance during the transition from vegetative growth to reproduction and senescence. For plant C, N stoichiometry, C content remained relatively stable, whereas N content varied significantly along the salinity gradient, resulting in a substantially higher plant C:N ratio in the late growth stage than in the early growth stage. Soil C:N ratio was the dominant factor influencing plant NSC and C, N stoichiometry in the early growth stage, driven by plant nitrogen demand; soil pH and electrical conductivity became dominant factors in the late growth stage, driven by soil salinization and pH conditions that affect nitrogen uptake and ion balance, indicating that the regulation of plant carbon and nitrogen metabolism by soil properties was stage−dependent.

**Conclusion:**

Our results revealed that *S. viridis* adapts to soil salinity through coordinated regulation of NSC components, carbon and nitrogen balance, and organ−specific allocation, with stage−specific responses to soil drivers. These findings provide a scientific basis for understanding plant adaptation to saline-alkaline environments, and offer practical guidance for species selection and ecological management for vegetation restoration in these regions.

## 1 Introduction

Soil salinization is one of the major abiotic factors limiting plant growth and development (Zhang et al., 2022; van Zelm et al., 2020). Currently, approximately 10.7% of the global land area is affected by salinization (Hassani et al., 2021), and this proportion is projected to increase under ongoing global change (Wang et al., 2003). Salt stress disrupts fundamental metabolic processes in plants through ionic toxicity and osmotic imbalance, directly constraining plant growth and development (Zhu, 2016). Also, salt stress can induce stomatal closure and damages the photosynthetic system of plant (Wang and Wang, 2026; Xue et al., 2021), thereby constraining plant carbon assimilation and directly affects the synthesis of non−structural carbohydrates (NSC) (Zhang et al., 2024). Meanwhile, it inhibits plant nitrogen uptake and utilization (Machado et al., 2025), further perturbing plant carbon and nitrogen metabolism (Cui et al., 2019). Therefore, unraveling how NSC, carbon (C), nitrogen (N) and their ratios respond to salt stress, and how these responses are coupled, is essential for understanding plant adaptation to saline environments.

NSC, composed primarily of soluble sugars and starch, represents the carbon storage form in plants and are critical for plant growth, metabolism and stress responses (Signori−Müller et al., 2021). Its dynamics serve as key indicators of plant carbon balance (Martínez−Vilalta et al., 2016). Generally, the contents of total NSC and its components exhibit distinct seasonal dynamics (Silvestro et al., 2024). Temperate deciduous trees rely on stored starch for bud break in the early growth stage, while in autumn photosynthates are preferentially allocated to stems and roots for storage, reflecting a distinct seasonal carbon allocation strategy (Hoch et al., 2003; Richardson et al., 2013). Also, the dynamics of total NSC and its components in different plant organs show contrasting patterns under stress (Huang et al., 2023; Nawaz et al., 2025). For instance, under drought and salt stress, *Eucommia ulmoides* seedlings exhibited contrasting responses in carbohydrate dynamics between coarse and fine roots: coarse roots showed decreased levels of soluble sugars and NSC, whereas fine roots displayed increased levels of soluble sugars and starch, reflecting functional partitioning in root carbon allocation under abiotic stress (Zhang et al., 2024). However, current knowledge of the differential dynamics of NSC and its components between aboveground and belowground organs remains limited. Moreover, while drought stress has received extensive attention, the response of plant NSC to salt stress remains elusive.

However, the allocation and turnover of plant NSC are not isolated processes but are tightly coupled with mineral nutrition (Zhou et al., 2025). Plant C, N stoichiometry reflects the balance between carbon assimilation and nitrogen acquisition and utilization (Liu et al., 2020). Carbon is the most fundamental element of life, forming the basis of organic compounds and participating in key metabolic reactions, from photosynthetic assimilation to respiration maintenance of growth and development (Dusenge et al., 2019). Nitrogen is a major constituent of genetic material and enzymes in organisms (Raven, 2012), and its content directly affects plant growth, development and metabolism (Fu et al., 2022; Chen et al., 2025). Plants have different physiological demands for elements at different life history stages (Rong et al., 2015) and can adapt to environmental changes by adjusting their elemental contents, resulting in dynamic differences in elemental stoichiometry. Consequently, plant C:N ratio serves as a key indicator of nutrient use efficiency and metabolic strategy (Zhang et al., 2020). Therefore, elucidating the coupling between plant NSC and C, N stoichiometry is critical for understanding how plants coordinate carbon metabolism with nutrient balance. However, current research on this interplay remains limited, with most studies focusing on factors such as water availability, nutrient addition or light (Xie et al., 2022; Liu et al., 2024). In contrast, the link between plant NSC and C, N stoichiometry under salt stress remains largely unexplored.

Studies have shown that plant NSC and C, N allocation are influenced by soil factors (Gu et al., 2024; Zhi et al., 2025). Soil water status governs the transport and storage of carbon assimilation products, and plants tend to increase carbon allocation to roots to enhance water acquisition under water deficit (Tariq et al., 2024). Soil salinity and electrical conductivity impose osmotic stress and ionic toxicity, prompting plants to allocate more carbon to the synthesis of osmotic regulators and root growth to maintain homeostasis (Li et al., 2023). Soil pH modulates element solubility and microbial community composition, thereby reshaping stoichiometric balances among plant organs (Adamczyk−Szabela and Wolf, 2022). These soil factors do not operate in isolation; rather, they interactively shape plant C, N stoichiometric adaptation strategies across habitats. Nevertheless, the primary drivers of plant carbon allocation and elemental stoichiometry under salt stress remain elusive. Moreover, current research has largely focused on a few common crops (e.g. wheat, sorghum) (Liang et al., 2017) and halophytes (e.g. *Phragmites australis*, *Suaeda glauca*, *Suaeda salsa*) (Jiao et al., 2020; Wang et al., 2024c; Sun et al., 2017), whereas the carbon allocation and stoichiometric responses of species commonly employed in saline−alkaline ecological restoration remain poorly understood, hindering a comprehensive assessment of interspecific differences in adaptive mechanisms.

*Setaria viridis* (L.) Beauv., an annual herbaceous plant and a common pioneer species in vegetation ecological restoration, exhibits strong adaptability and has been widely employed in ecosystem restoration for bio−soil stabilization (Wang et al., 2024b). The Xiaokai River irrigation area, situated in the hinterland of the Yellow River Delta, China, represents one of the large−scale irrigation areas that diverts water from the Yellow River. Due to the flat topography, shallow water table and high evaporation, soil salinization constitutes a prominent environmental challenge in this region, forming a natural gradient from very low to very high salinity along the irrigation area from south to north as elevation decreases (Jiang et al., 2023). We measured the plant NSC content, and C, N stoichiometry in aboveground and belowground parts of *S. viridis* in different growth stages, along with soil pH, electrical conductivity, C and N, across a salinity gradient in the Xiaokai River irrigation area. We hypothesized that (i) soil salinity significantly alters the dynamics of plant NSC and its components (soluble sugars and starch), as well as plant C, N stoichiometry, in both aboveground and belowground parts; (ii) plant NSC and its components are closely coupled with plant C, N stoichiometry in aboveground and belowground parts, reflecting coordinated resource allocation strategies under salinity stress; (iii) the relative importance of soil factors in regulating plant carbon and nitrogen metabolism shifts with plant growth stages.

## 2 Materials and methods

### 2.1 Site description

The Xiaokai River irrigation area (117°42′-118°04′ E, 37°17′-38°03′ N) diverts unembanked water from the Yellow River, a process that inevitably introduces sediment and results in continuous siltation within the irrigation area. Therefore, the main canal was designed with reference to historical data on the Yellow River bed elevation and water level upstream of the regulating sluice, adopting a high−slope, long−distance sediment transport approach. The main canal of Xiaokai River is 91.5 km in length, including a 51.3 km sediment transport channel, a 4.16 km sedimentation basin, and a 36.04 km water conveyance channel.

The Xiaokai River irrigation area is situated in the accumulation plain of the central Yellow River Delta, China. The region experiences a temperate monsoon climate characterized by four distinct seasons and synchronous rain and heat. Precipitation is highly variable and unevenly distributed, with a mean annual precipitation of 575.2 mm and a mean annual temperature of 12.3 °C. The average annual frost−free period is 210 days, and annual sunshine duration ranges from 2400 to 2700 hours. Soils are predominantly fluvo−aquic, and the surface soil texture comprises four main categories: sandy soil, sandy loam, clay loam, and clay.

### 2.2 Experimental design

**Figure 1:**
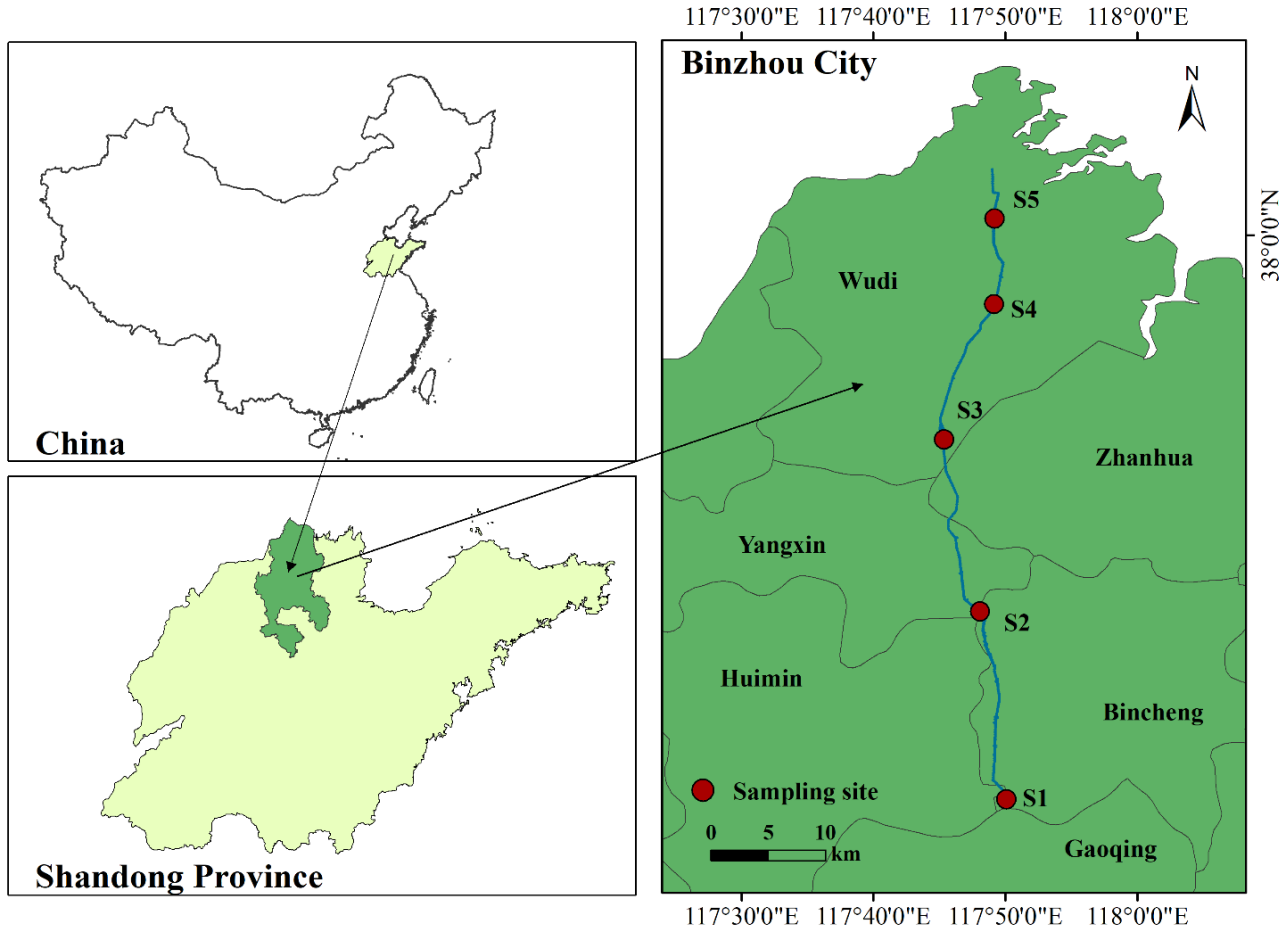
Location of the study area and distribution of sampling sites. S1, very low salinity; S2, low salinity; S3, medium salinity; S4, high salinity; S5, very high salinity.

**Figure 2:**
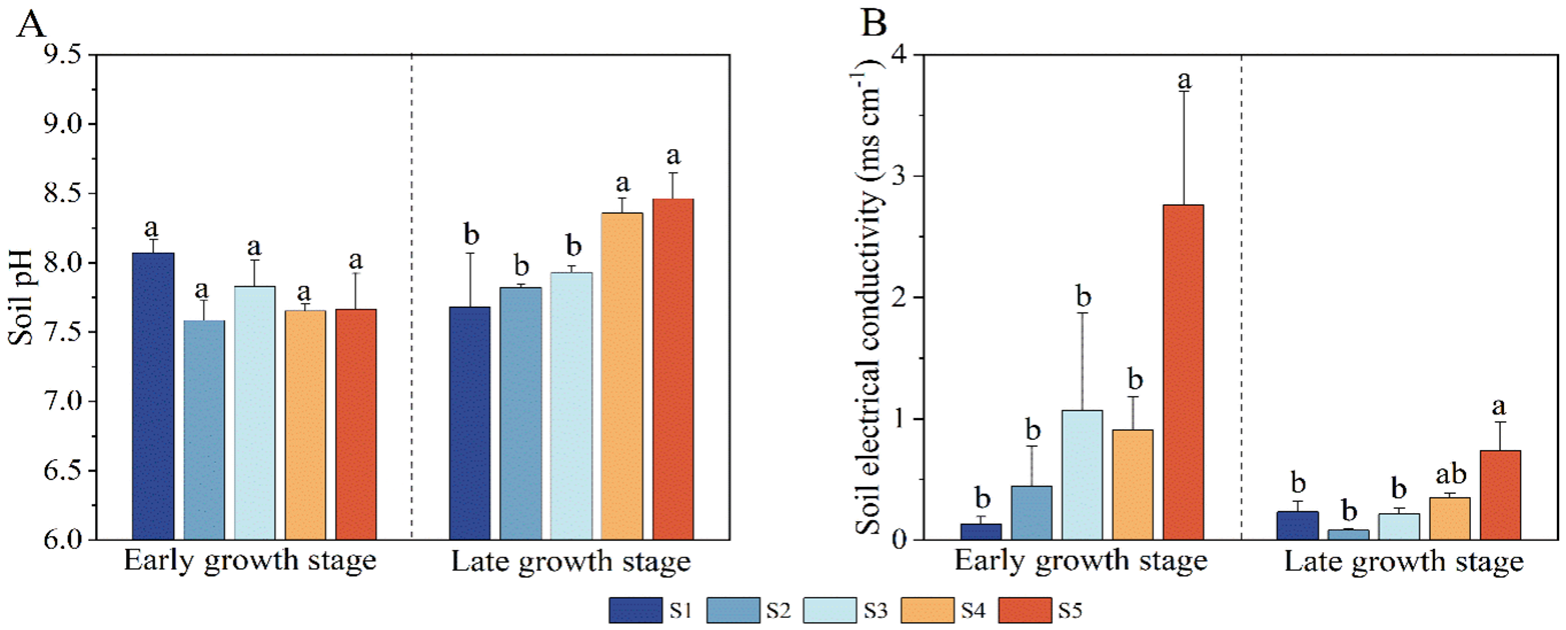
Soil pH and electric conductivity across sampling sites along the soil salinity gradient. **(A)** Soil pH; **(B)** soil electrical conductivity. Different lowercase letters indicate significant differences amongst plots within the same part and growth stage.

Plant and soil sampling was conducted in late-May and mid−September of 2021. Late May corresponds to the vegetative growth stage of *S. viridis*, and mid−September corresponds to the late fruiting to maturation stage (Chen and Phillips, 2006). We defined these two time points as the early growth stage and the late growth stage, respectively. Five sampling sites were established from south to north along the Xiaokai River irrigation area, spanning from the upper to the lower reaches of the main canal (**Figure 1**). The five sites were located along the main canal at approximately 0.0, 26.5, 52.5 (within the sedimentation basin), 69.4, and 81.4 km from the irrigation source, with two sites upstream and two downstream of the sedimentation basin. Reflecting the decrease in elevation, the degree of salinity increased gradually across the five sampling sites (S1: very low salinity; S2: low salinity; S3: medium salinity; S4: high salinity; S5: very high salinity). Site−specific soil pH and electrical conductivity are shown in **Figure 2**. The higher soil electrical conductivity in the early growth stage than in the late growth stage is due to spring evaporation−induced salt accumulation and summer rainfall leaching, a typical hydrological regime in the Yellow River Delta. At each site and growth stage, three to five quadrats (2 × 2 m) were randomly established at inter-quadrat distances greater than 30 m. Within each quadrat, approximately 30 individual plants were collected and pooled into one sample (aboveground and belowground parts separated). Each quadrat served as one biological replicate, yielding 3 to 5 replicates per site for each tissue and growth stage. Additionally, one soil core (0-20 cm depth) was taken from each quadrat. Plant samples were oven−heated at 105 °C for 1 h to deactivate enzymes, then dried at 65 °C to constant weight. Soil samples were air−dried and remaining roots and stones were manually removed.

For C and N analyses, plant and soil samples were ground to a fine powder using a ball mill (WS-MM301; Retsch, Haan, Germany) and passed through a 0.15 mm sieve. Total C and N contents were determined by combustion using an elemental analyzer (vario MACRO cube, Germany). Soluble sugars were extracted from sample powder with 80 % ethanol. The residue was then dried at 75 °C and digested with α-amylase to hydrolyze starch into glucose. Both soluble sugar and starch contents were measured using the phenol-sulfuric acid method, and total NSC content was calculated as the sum of the two components. Soil pH was measured using a glass electrode pH meter (S40, Mettler Toledo, Switzerland) in a 1:2.5 soil-to-water suspension after stirring for 2 min standing for 30 min. Soil electrical conductivity was determined using a conductivity meter (DDS-11A, Leici, China) on a 1:5 soil-water extract, with measurements taken in the supernatant after equilibration.

### 2.3 Data analysis

One−way analysis of variance (ANOVA) was used to analyze differences in plant NSC content and C, N stoichiometry in plant samplings along the salinity gradient. Two-way ANOVA was performed to test the effects of soil salinity, growth stage, and their interaction on plant NSC content and C, N stoichiometry in plant samples (**Supplementary Tables 1**, **2**). Independent samples t−tests were conducted to compare plant NSC content and C, N stoichiometry between aboveground and belowground parts within the same growth stage, as well as between stages for the same plant tissue. Pearson’s correlation analysis was employed to examine relationships between plant NSC content and C, N stoichiometry in both aboveground and belowground parts. Above statistical analyses were performed using SPSS (version 21.0, SPSS Inc., Chicago, IL, USA). Redundancy analysis (RDA) was conducted to assess the relationships between plant NSC content and C, N stoichiometry and soil properties using CANOCO for Windows (version 5.0, Ithaca, NY, USA), with forward selection applied to identify significant soil factors. The *P* values and relative contributions of each soil factor, derived from Monte Carlo permutation tests, are provided in **Supplementary Table 3**.

## 3 Results

### 3.1 Dynamics of plant NSC and its components along the soil salinity gradient

**Figure 3:**
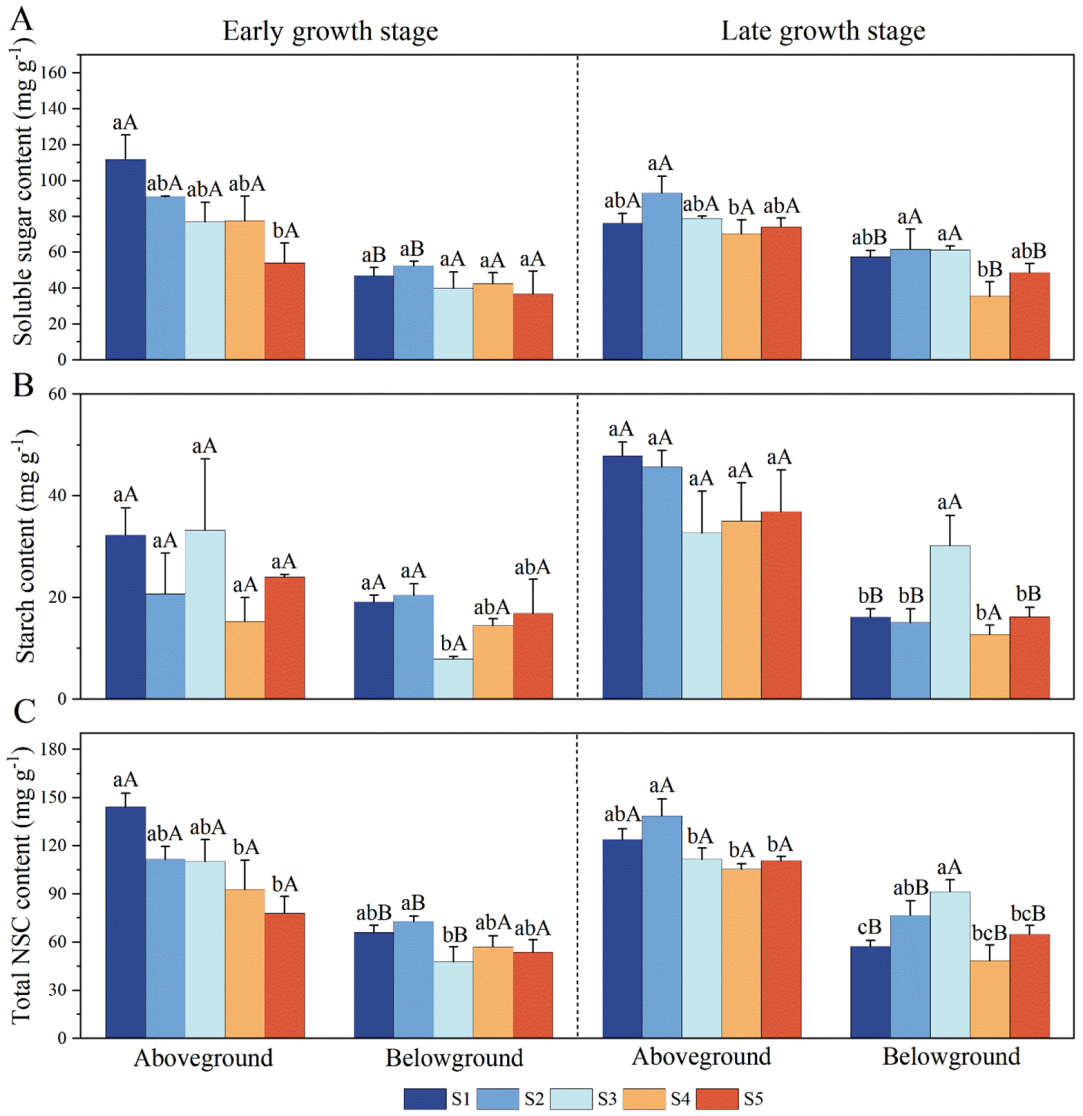
Dynamics of plant soluble sugar, starch and total NSC content in response to soil salinity and growth stage. **(A)** Soluble sugar content; **(B)** starch content; **(C)** total NSC content. Different uppercase letters indicate significant differences between aboveground and belowground parts of the same growth stage. Different lowercase letters indicate significant differences amongst plots within the same part and growth stage. The same below.

The plant NSC and its component contents in aboveground and belowground parts displayed distinct dynamics along the soil salinity gradient, with significant interactions between salinity and growth stage (**Figure 3**; **Supplementary Figures 1**; **Supplementary Table 1**). In the early growth stage, aboveground soluble sugar content remained stable initially and then decreased, whereas belowground showed no significant change with increasing soil salinity. In the late growth stage, aboveground soluble sugar content first increased and then decreased, while belowground remained stable initially and then decreased along the salinity gradient. Aboveground starch content showed no significant changes in both growth stage; whereas belowground starch exhibited opposite trends between growth stages: first decreased and then increased in the early growth stage, but first increased and then decreased in the late growth stage along the salinity gradient. Along the salinity gradient, aboveground NSC content decreased in the early growth stage, but it remained stable initially and then decreased in the late growth stage; belowground NSC content followed the same trend as starch. Plant NSC and its component contents in both aboveground and belowground parts also varied with growth stages (**Supplementary Figure 1**). The aboveground starch content in the early growth stage was significantly lower than that in the late growth stage.

### 3.2 Dynamics of plant C, N stoichiometry along the soil salinity gradient

**Figure 4:**
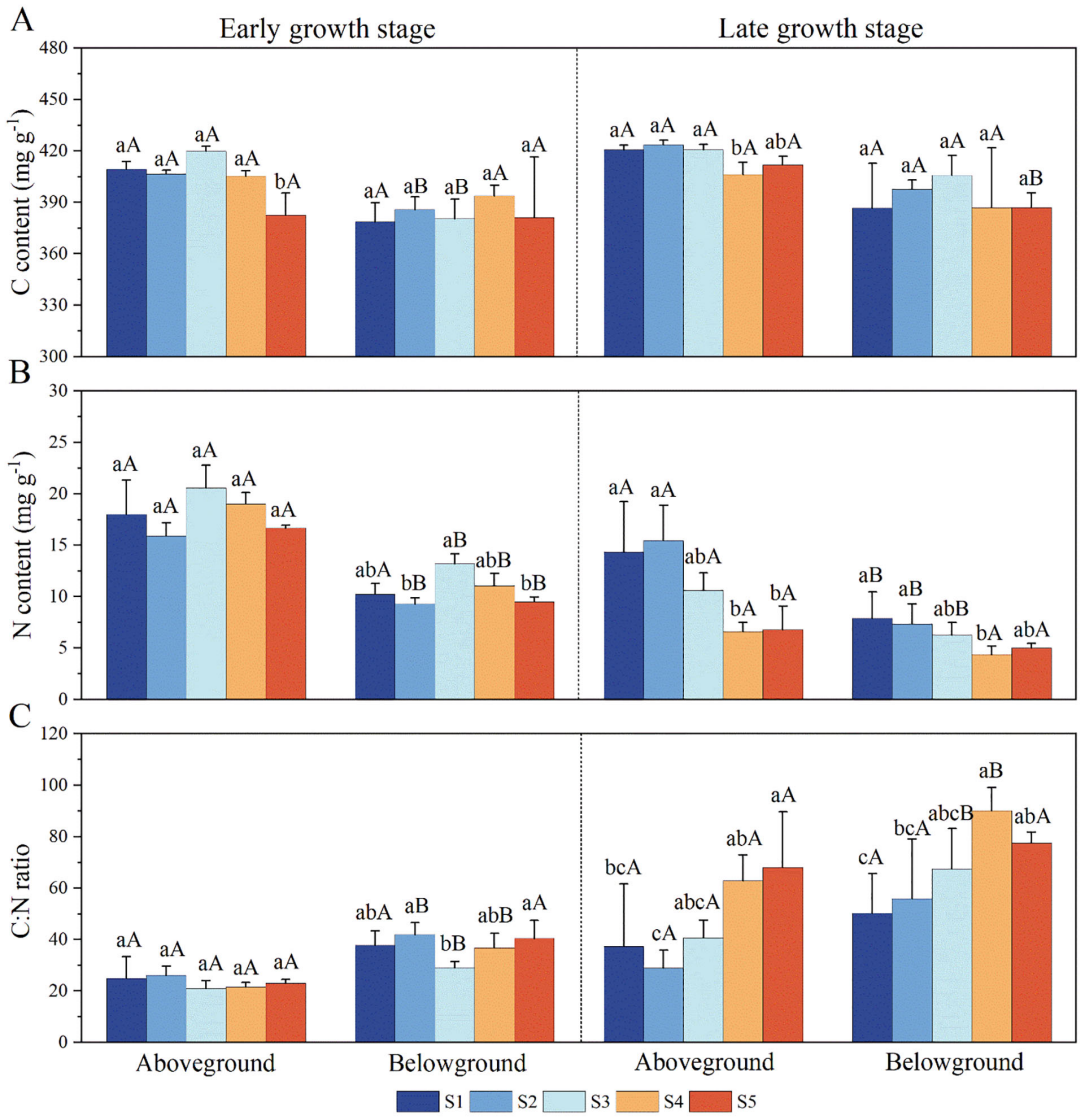
Dynamics of plant C, N stoichiometry in response to soil salinity and growth stage. **(A)** C content; **(B)** N content; **(C)** C:N ratio.

The plant C, N stoichiometry in aboveground and belowground parts exhibited distinct dynamics along the soil salinity gradient, with significant interactions between salinity and growth stage (**Figure 4**; **Supplementary Figures 2**; **Supplementary Table 2**). Plant C content shows similar patterns in both growth stages: aboveground C content remained stable initially and then decreased significantly, whereas belowground C content showed no significant change with increasing soil salinity. In the early growth stage, aboveground N content showed no significant variation, whereas belowground N content first increased and then decreased along the salinity gradient. In the late growth stage, both aboveground and belowground N contents remained stable initially and then decreased with increasing soil salinity. In the early growth stage, aboveground C:N ratio showed no significant change, whereas belowground C:N ratio first decreased and then increased along the salinity gradient. In the late growth stage, both aboveground and belowground C:N ratios increased with increasing soil salinity. Plant C, N stoichiometry in both aboveground and belowground parts also varied with growth stages. Aboveground C content in the early growth stage was significantly lower than that in the late growth stage (**Supplementary Figure 2**). Aboveground and belowground N contents in the early growth stage were significantly higher than those in the late growth stage. Aboveground and belowground C:N ratios in the early growth stage were significantly lower than those in the late growth stage.

### 3.3 Relationships between plant NSC and C, N stoichiometry in aboveground and belowground parts

**Figure 5:**
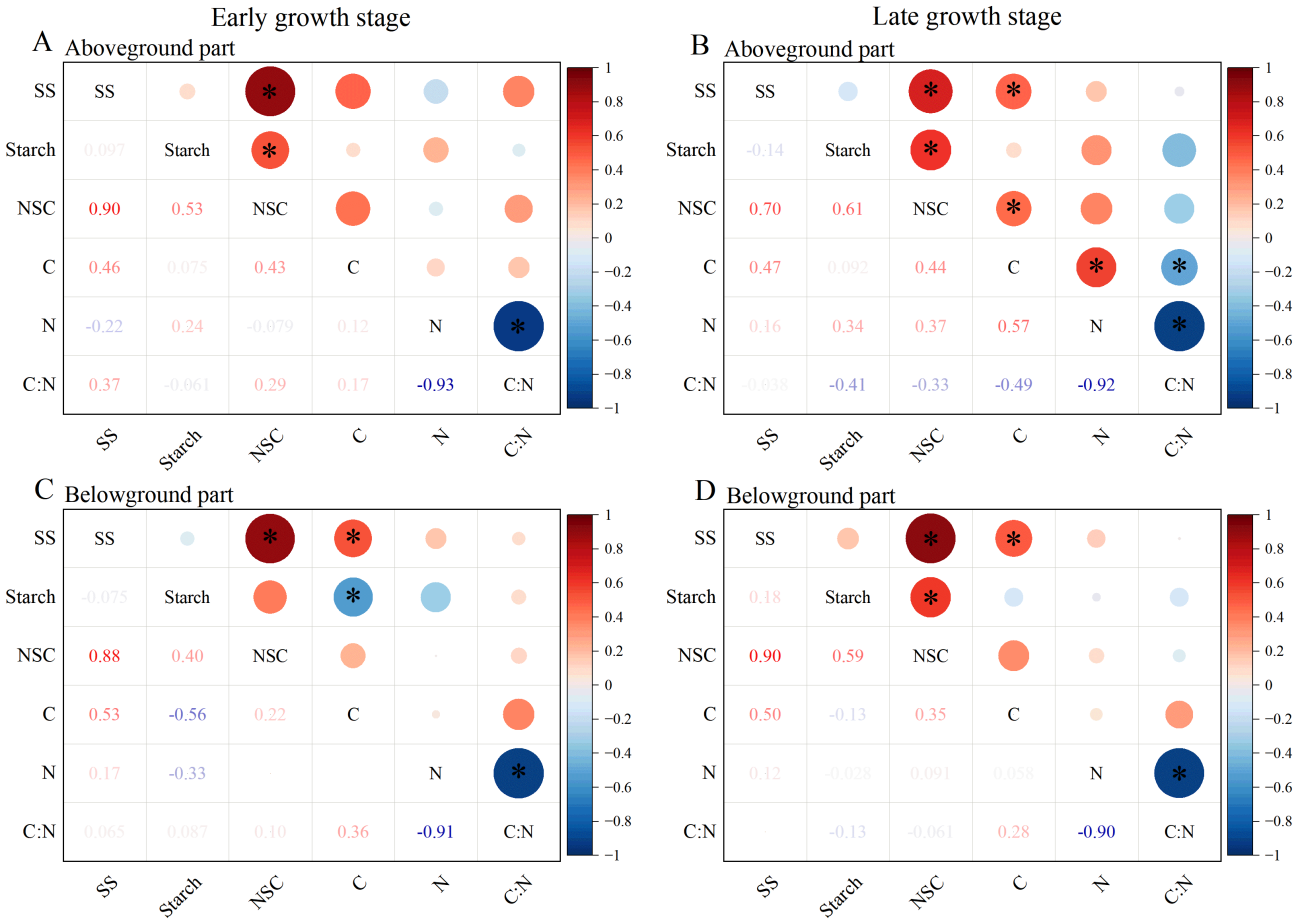
Relationships between plant NSC and C, N stoichiometry in aboveground and belowground parts during the early **(A, C)** and late **(B, D)** growth stages. *represents significant correlations at the 0.05 level.

Plant NSC and C, N stoichiometry differed between plant tissues and growth stages (**Figure 5**). Across different plant tissues, aboveground NSC was positively correlated with soluble sugar and starch contents, whereas C:N ratio showed a negative correlation with N content. In contrast, the belowground part showed positive correlations of starch with NSC and C content in both early and late growth stages, and C:N ratio remained negatively correlated with N content. In the early growth stage, NSC in both aboveground and belowground parts was strongly positively correlated with soluble sugar content and moderately positively correlated with starch content, while C:N ratio was negatively correlated with N content. In the late growth stage, NSC in both aboveground and belowground parts still exhibited a strong positive correlation with soluble sugar content and a moderate positive correlation with starch content. Different from the early growth stage, soluble sugar showed a moderate positive correlation with C content, while C:N ratio remained negatively correlated with N content.

### 3.4 Relationships between plant NSC and C, N stoichiometry and soil properties

**Figure 6:**
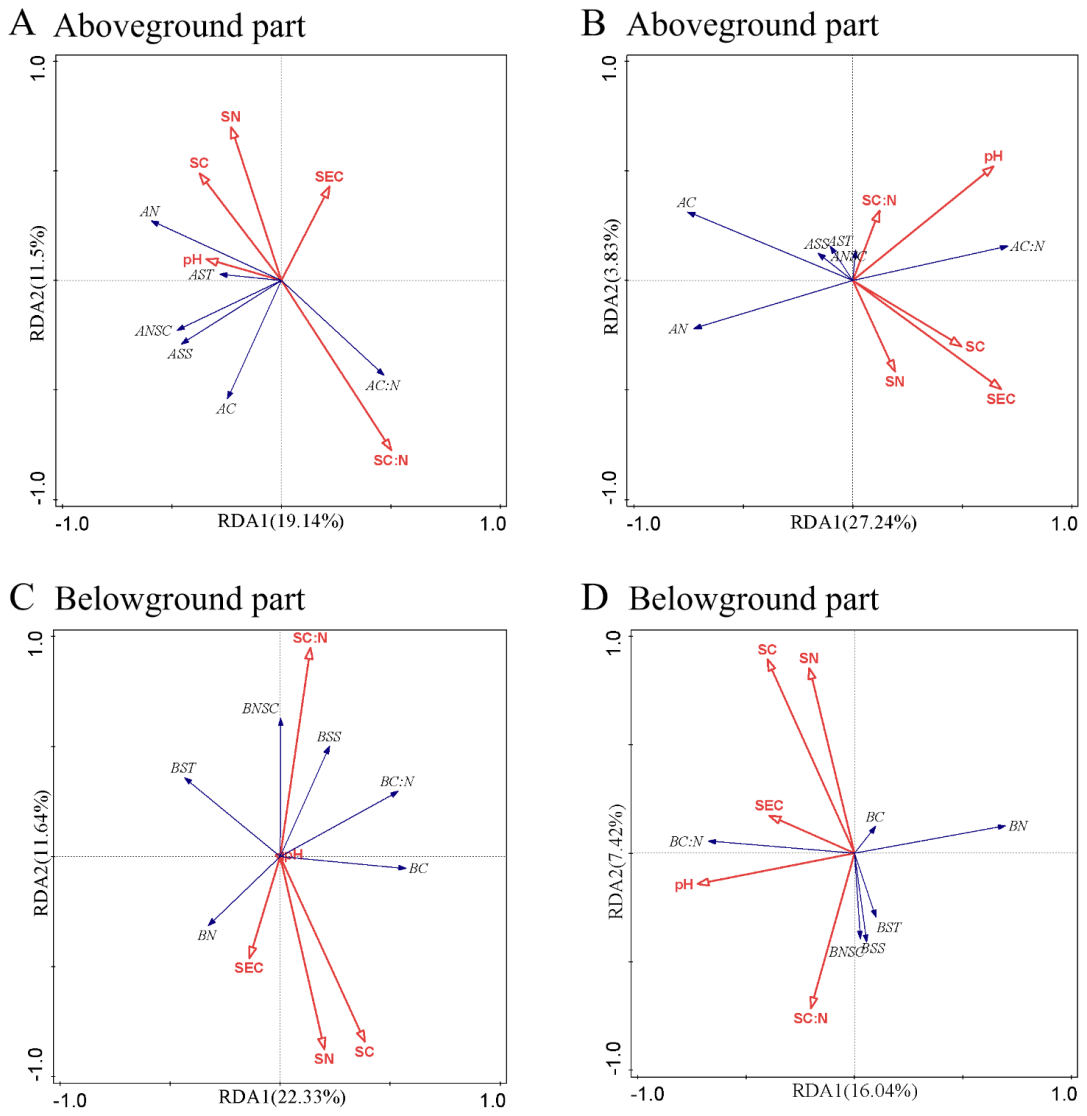
RDA-ordination biplot of plant NSC and C, N stoichiometry in above- and belowground parts and soil properties during the early **(A, C)** and late **(B, D)** growth stages. AC/BC, aboveground/belowground C content; AN/BN, aboveground/belowground N content; AC:N/BC:N, aboveground/belowground C:N ratio; ANSC/BNSC, aboveground/belowground NSC content; AST/BST, aboveground/belowground starch content; ASS/BSS, aboveground/belowground soluble sugar content; SC, soil C content; SN, soil N content; SC:N, soil C:N ratio; pH, soil pH; SEC, soil electrical conductivity.

The RDA ordination biplot showed that the primary soil factors driving plant NSC and C, N stoichiometry varied with plant tissue and growth stage (**Figure 6**; **Supplementary Table 3**). In the early growth stage, soil C:N ratio was the dominant factor influencing aboveground parts, explaining 35.5% of the variation. In the late growth stage, soil electrical conductivity and pH emerged as the dominant determinants for aboveground parts, accounting for 41.6% and 29.6% of the variation, respectively. For belowground parts, soil C:N ratio also dominant in the early growth stage, explaining 55.7% of the variation; whereas soil pH became the dominant factor in the late growth stage, explaining 36.9% of the variation.

## 4 Discussion

### 4.1 Dynamics of plant NSC and its components along the soil salinity gradient

The responses of plant NSC and its components to salt stress were tissue−specific and stage−dependent. Aboveground parts exhibited significant changes in soluble sugar content along the salinity gradient, whereas starch content remained relatively stable, indicating that the carbohydrate dynamics of aboveground parts were primarily driven by soluble sugars. In contrast, belowground starch varied dynamically, and soluble sugars decreased only under high salinity in the late growth stage. These distinct patterns likely reflect different functional demands between organs. Aboveground parts require rapid osmotic adjustment to maintain photosynthesis, and soluble sugars act as fast−responding osmoregulatory compounds (Afzal et al., 2021; Yang et al., 2016). Belowground parts, chronically exposed to soil salinity and persistent ionic stress, depend more on starch storage and remobilization to buffer long−term fluctuations in carbon supply and demand (Thalmann and Santelia, 2017). Moreover, plant NSC and its components in aboveground part were consistently more abundant than those in belowground parts in both early and late growth stages. This is probably because aboveground parts are the primary sites of photosynthesis. However, it is important to note that as an annual plant, the root system of *S. viridis* functions primarily as a turnover organ for resource uptake and temporary storage rather than as a long-term carbon reservoir, in contrast to perennial plants (Gonzalez-Paleo et al., 2024). Consequently, plant NSC tends to accumulate preferentially in shoots to support rapid growth and reproduction. Together with a growth strategy that prioritizes aboveground growth, plant NSC content were consistently higher aboveground than belowground (Wang et al., 2023). This pattern is consistent with observations in other annual plants, such as upland rice and grain amaranth (Castrillón-Arbeláez et al., 2012; Bezerra et al., 2021).

Stage dependence was evident in how soluble sugar and starch responses varied with growth stage. In the early growth stage, aboveground soluble sugar content decreased significantly only under extremely high salinity, suggesting that during the vegetative stage plants prioritize leaf growth and structural development (Monson et al., 2022; Wang et al., 2024a). In the late growth stage, aboveground soluble sugar first increased and then decreased along the salinity gradient, implying that moderate salt stress may stimulate soluble sugar accumulation either to protect reproductive organs or to serve as an osmoregulatory compound (Pilon et al., 2019; Munns and Tester, 2008). Belowground starch displayed opposite trends between the two growth stages: it first decreased then increased with salinity in the early growth stage, but first increased then decreased in the late growth stage. The decrease in starch under low salinity during the early growth stage may reflect root starch mobilization, providing carbon skeletons and energy for the synthesis of osmoregulatory compounds. The increase in starch under high salinity, by contrast, may result from severe inhibition of root metabolic activity that slows starch degradation, while aboveground parts continue exporting photosynthates to the roots, leading to starch accumulation in roots (Ludewig and Flügge, 2013). In the late growth stage, the increase in starch at low salinity probably reflects greater carbon partitioning to root storage during reproduction. Conversely, the decrease under high salinity is likely caused by severely impaired aboveground photosynthetic capacity and reduced carbon transport to roots (Hartmann et al., 2020b). Aboveground starch remained insensitive to salt stress at both growth stages, but its content was significantly lower in the early growth stage than in the late growth stage, which may be associated with energy reserves required for seed set and senescence before plant dieback (MacNeill et al., 2017).

In this study, plant NSC and its component contents were significantly higher under low salinity (S1-S2) than under high salinity (S4-S5), suggesting that when carbon resources are abundant, plants sustain higher carbon turnover and storage. Under high salinity, photosynthetic carbon assimilation is constrained, and substantial carbon are consumed for osmotic adjustment and ion transport. As a result, soluble sugar and NSC contents decline with increasing salinity, a trend that is more pronounced in aboveground parts. This result contrasts with the drought−induced NSC dynamics in woody plants reported by Hartmann et al. (2013a), highlighting a fundamental divergence in stress response strategies between herbaceous and woody plants. Herbaceous plants rely more on immediate carbon supply from source organs and osmotic regulation via soluble sugars, whereas woody plants tend to mobilize long−term carbon reserves (Martínez−Vilalta et al., 2016; Wang and Wang, 2025).

The stage-dependent shift from high soluble sugars in the early growth stage to increased starch in the late growth stage reflects a reallocation of carbon from immediate utilization to reserve storage (Gibon et al., 2009; Dong and Beckles, 2019). This pattern indicates that as the plant transitions from vegetative growth to reproduction and senescence, carbon partitioning shifts toward starch accumulation as a longer−term reserve. Under low salinity, plants have abundant carbon resources, which are allocated not only to growth and development but also to surplus carbon storage, resulting in both high carbon turnover and high storage levels. Under high salinity, by contrast, carbon resources become limited, leading to low turnover and minimal storage. In this study, the variation of plant NSC and its components along the salinity gradient conforms to the optimal partitioning theory (Liu et al., 2021), which posits that plants balance trade-offs between resource acquisition and stress tolerance. When conditions are favorable, plants invest preferentially in aboveground parts to compete for light and space; when stress intensifies, they increase investment in belowground parts to acquire nutrients or shift toward carbon storage to ensure survival (Lemoine et al., 2013; De Battisti et al., 2020).

### 4.2 Dynamics of plant C, N stoichiometry along the soil salinity gradient

The responses of plant C, N stoichiometry to salt stress were also tissue−specific and stage-dependent. For plant C content, aboveground parts decreased significantly only under extremely high salinity, whereas belowground parts showed no significant change across salinity gradient. This suggests that salt stress exerts only a limited effect on the total plant carbon pool, which becomes evident only when high salinity severely impairs photosynthesis. This result contrasts with the pronounced changes in NSC components (soluble sugars and starch), indicating that carbon reallocation among different fractions is more sensitive than alterations in total carbon pool size. The response of aboveground C content to salt stress was significantly stronger in the early growth stage than in the late growth stage, consistent with the view that carbon assimilation is constrained at the onset of stress (Lawlor and Cornic, 2002). Specifically, salt−induced stomatal closure and reduced mesophyll conductance limit carbon supply from source organs, while increased carbon consumption for osmoregulatory compounds synthesis may lead either to transient carbon accumulation at the source end or to impeded carbon allocation to sink organs (Chaves et al., 2009).

In the early growth stage, belowground N content first increased and then decreased with increasing salinity, whereas aboveground N content showed no significant change. This pattern may reflect active nitrogen uptake by roots under moderate salt stress to support the synthesis of nitrogen-containing osmoregulatory compounds such as proline and betaine, or to meet the protein demand for repairing salt−induced damage (Ashraf and Foolad, 2007; Li et al., 2022; Nazir et al., 2023). Under high salinity, nitrogen uptake is suppressed, leading to a decline in N content. In the late growth stage, belowground N content decreased significantly with increasing salinity. Under high salinity, root ion uptake is inhibited, and plants may sustain photosynthesis by reducing nitrogen storage in roots and enhancing nitrogen transport to aboveground parts (Zhang et al., 2023). In both the early and late growth stages, C content showed little variation along the salinity gradient, whereas N content exhibited a marked decline. This may be because structural carbon in plant tissues is less affected by environmental fluctuations than functional nitrogen (Xing et al., 2021).

In the early growth stage, the belowground C:N ratio first decreased and then increased with salinity, a trend opposite to that of N content, indicating that under moderate salt stress roots actively increase N uptake to lower the C:N ratio and sustain metabolic activity. In the late growth stage, the C:N ratio in both aboveground and belowground parts increased steadily with salinity, a pattern consistent with observations by Zhou et al. (2021) in plants from inland riverine wetlands of northwest China. This increase arises primarily because salt stress suppresses N uptake while carbon assimilation declines only modestly, creating a relative carbon surplus. This result reflects a conservative metabolic strategy of *S. viridis* under salt stress during the late growth stage, prioritizing carbon reserve over biomass growth. Moreover, regardless of growth stage, the plant C:N ratio was consistently higher belowground than aboveground, confirming that roots serve as carbon sinks that maintain greater carbon reserves under salt stress, thereby providing energy for ion compartmentation, osmotic adjustment, and oxidative stress repair (Erel et al., 2020; Li et al., 2023).

### 4.3 Relationships between plant NSC and C, N stoichiometry in aboveground and belowground parts

In this study, the relationships between plant NSC and C, N stoichiometry varied with plant tissues and growth stages. In the early growth stage, NSC accumulation was primarily driven by soluble sugars, accompanied by starch synthesis, to meet the demands of rapid photosynthetic carbon storage and cell division and expansion during the early growth stage (Liu et al., 2023). The increase in aboveground C content depended directly on photosynthetic NSC accumulation, while plant N content showed a strong negative correlation with the plant C:N ratio, reflecting a nitrogen−priority allocation strategy. This strong negative correlation persisted across all tissues and growth stages, indicating that N plays a central role in modulating the C:N ratio throughout the entire life cycle. This is likely because when N supply is abundant, plants maintain a lower C:N ratio and allocate resources preferentially to vegetative growth and carbon storage; when N becomes limiting or C become relatively enriched, the C:N ratio increases, promoting the transition to reproductive growth (Diaz et al., 2008; Shi et al., 2026).

In the late growth stage, the elevated plant C:N ratio promotes the transition to reproductive growth, consistent with observations by Amoah et al. (2026). Aboveground starch accumulation declines, while the coupling between plant C and N becomes tighter, with N increasingly coordinated with starch metabolism to support reproductive organ development. NSC accumulation remains similar to that in the early growth stage. Belowground, the coupling between plant C and N weakens, and more carbon is stored as starch to maintain root survival. As expected, plant NSC content was positively correlated with both soluble sugar and starch contents across growth stages and tissues. From the early to late growth stage, the correlation between soluble sugars and NSC weakened aboveground, whereas the correlation between starch and NSC strengthened. This pattern suggests that under prolonged salt stress, carbon partitioning favors the accumulation of starch over soluble sugars, thereby reducing the risk of osmotic potential fluctuations (Basu et al., 2007). This direction of response is opposite to the starch to sugar pattern observed under short−term stress (Thalmann et al., 2016), reflecting a strategy shift from rapid osmotic adjustment under acute stress to steady−state storage protection under chronic stress (Jumpa et al., 2022). Belowground, the relationship between NSC and soluble sugars remained more stable than aboveground, but the correlation between NSC and starch increased markedly in the late growth stage, suggesting that root carbon reserves are progressively enhanced as salt stress persists.

### 4.4 Relationships between plant NSC and C, N stoichiometry and soil properties

In this study, plant NSC and its components, as well as plant C, N stoichiometry, were influenced by soil properties, with different factors dominating at different growth stages. As an annual plant, *S. viridis* adopts a fast−growth strategy in the early growth stage, prioritizing aboveground biomass accumulation, carbon−nitrogen metabolism, and nutrient uptake. This period is a critical for plants to establish efficient resource−use strategies, with physiological responses centered on C and N metabolism and nutrient acquisition (Poorter et al., 2012; Monson et al., 2022). Consequently, soil C:N ratio emerged as the dominant factor, reflecting nitrogen availability and organic matter decomposition rate (Ostrowska and Porebska, 2015). This occurred despite higher soil electrical conductivity in the early growth stage, likely because the plant’s high nitrogen demand overrode the direct effects of salinity. At this stage, plants grow rapidly and construct their tissues, exhibiting high N demand, and C and N metabolism is driven primarily by nutrient availability. *S. viridis* acquires nutrients from the soil, consistent with the strong dependence of seedlings on soil nutrient supply (Martini et al., 2020). This indicates that the direct contact of roots with soil enables a more rapid response to soil properties, and also reflects that belowground stoichiometry is more tightly coupled with soil conditions (Yin et al., 2021). When soil C:N ratio is low, aboveground N content increases and the C:N ratio decreases, accompanied by corresponding changes in NSC accumulation patterns. Under ample nitrogen supply, plants allocate photosynthates preferentially to growth rather than storage, with concomitant shifts in the partitioning of soluble sugars and starch. During the early growth stage, roots function primarily in nutrient uptake and temporary storage. Soil C:N ratio directly regulates root nitrogen uptake and carbon partitioning through its influence on nitrogen availability. A low soil C:N ratio promotes root nitrogen accumulation, which in turn lowers the root C:N ratio and shifts the balance between soluble sugars and starch within NSC, directing more carbon toward osmotic regulation and root growth (Yang et al., 2016; Tariq et al., 2024; Zhou et al., 2025).In the late growth stage, *S. viridis* shifts to a stress tolerant life history strategy, prioritizing reproduction and stress resistance over further vegetative growth (Cho et al., 2025). By this time, the plant is in the late fruiting to senescent stage, and its demand for soil nitrogen declines substantially. Prolonged salt exposure leads to progressive accumulation of Na^+^ and Cl^-^ in plant tissues, while elevated soil pH further reduces nutrient availability. Although absolute soil electrical conductivity declined in the late growth stage, the plant’s reduced nitrogen demand and increased sensitivity to ionic stress made soil pH and electrical conductivity the dominant factors. Consequently, correlations between soil elemental stoichiometry and plant NSC and C, N stoichiometry (both aboveground and belowground part) weakened considerably, whereas soil pH and electrical conductivity become the dominant drivers. Physiological activity shifts from nitrogen limitation to stress response, and the direct regulatory role of soil stoichiometry diminishes. This transition from nutrient driven to stress driven regulation reflects a fundamental trade−off in annual plants, whereby early investment in growth is followed by late investment in stress tolerance and reproductive allocation. Soil electrical conductivity reflects soil salinity or soluble ion content, while soil pH influences nutrient availability and microbial community composition (Adamczyk-Szabela and Wolf, 2022; Ndabankulu et al., 2022). The dominance of both factors in the late growth stage indicates that aboveground C and N metabolism is then regulated by a combination of ionic stress, nutrient balance and microbial activity rather than by nutrient supply alone.

### 4.5 Limitations of this study

Several limitations should be acknowledged in this study. First, aboveground or belowground biomass was not measured. Consequently, we could not calculate total pools of plant C, N, and NSC. The present study relied on mass−based concentration data, which reflect physiological responses per unit mass but do not provide absolute quantities. Future studies should integrate content and biomass data to fully resolve carbon allocation strategies. Second, only one soil core was collected from each quadrat. Given the potential spatial heterogeneity of saline and alkaline soils, this approach may not fully capture within−quadrat variability. Although the low standard errors of soil pH and electrical conductivity within each site suggest that site−level patterns were reliably detected, future studies should adopt composite sampling to better account for local heterogeneity. Third, this study was conducted within a single irrigation system. Whether the observed patterns can be generalized to other saline-alkaline regions or broader environmental gradients warrants further investigation.

## 5 Conclusion

Our results reveal that plant NSC and its components, C, N stoichiometry and their determining soil properties varied with plant tissues and growth stages. In this study, plant NSC content was consistently higher in aboveground than in belowground across growth stages, indicating a growth−prioritized carbon strategy. Soluble sugars dominated carbon partitioning and utilization, whereas starch played a more prominent role in carbon storage during the late growth stage than in the early growth stage, reflecting a stage−dependent shift from energy supply to reserve assurance in plant carbon metabolism. Also, the relationships between plant C and N differed among plant tissues and growth stages. Plant C content remained relatively stable, whereas plant N content was sensitive to salinity, leading to a marked increase in plant C:N ratios in the late growth stage, which reflects an adaptive adjustment of resource investment to meet the demands of reproductive growth. Soil properties regulated plant C and N metabolism in a stage−dependent manner: soil C:N ratio dominated in the early growth stage, driven by plant nitrogen demand; in the late growth stage, soil pH and electrical conductivity became the dominant factors, driven by soil salinization and pH conditions that affect nitrogen uptake and ion balance. Overall, this study underscores the importance of growth stage in mediating plant and soil interactions under salinity, and provides practical guidance for ecological restoration in saline-alkaline ecosystems. Future research should incorporate a wider range of environmental factors and integrate stoichiometric analyses of litter and rhizosphere components to elucidate the environmental response thresholds of plant resource strategies in saline−alkaline regions and how these strategies respond to variations in the rhizosphere micro−environment.

## Data Availability

The raw data supporting the conclusions of this article will be made available by the authors, without undue reservation.
