# Supplementary material for: Stage-dependent shifts in non-structural carbohydrates and C, N stoichiometry in Setaria viridis along a salinity gradient in the Xiaokai River irrigation area of the Yellow River Delta, China
Source: Front Plant Sci. 2026 Jul 15;17:1880438. doi: 10.3389/fpls.2026.1880438 (PMC13416748; doi:10.3389/fpls.2026.1880438)
Supplement: Supplementary file 1 [file DataSheet1.docx]

Supplementary Material

Table S1 Results of two‑way ANOVA for soluble sugar, starch, and total NSC contents in different plant parts

| Components | Growth part | Source of variation | DF | *F* | *P* |
| --- | --- | --- | --- | --- | --- |
| Soluble sugar | Aboveground | Salinity | 4 | 4.333 | 0.008 |
|  |  | Growth stage | 1 | 0.457 | 0.505 |
|  |  | Salinity × Growth stage | 4 | 2.922 | 0.040 |
|  | Belowground | Salinity | 4 | 2.108 | 0.107 |
|  |  | Growth stage | 1 | 1.739 | 0.198 |
|  |  | Salinity × Growth stage | 4 | 1.393 | 0.263 |
| Starch | Aboveground | Salinity | 4 | 1.084 | 0.384 |
|  |  | Growth stage | 1 | 11.974 | 0.002 |
|  |  | Salinity × Growth stage | 4 | 0.884 | 0.487 |
|  | Belowground | Salinity | 4 | 0.798 | 0.537 |
|  |  | Growth stage | 1 | 1.332 | 0.259 |
|  |  | Salinity × Growth stage | 4 | 6.219 | 0.001 |
| Total NSC | Aboveground | Salinity | 4 | 5.570 | 0.002 |
|  |  | Growth stage | 1 | 2.760 | 0.109 |
|  |  | Salinity × Growth stage | 4 | 2.232 | 0.093 |
|  | Belowground | Salinity | 4 | 3.267 | 0.026 |
|  |  | Growth stage | 1 | 3.561 | 0.070 |
|  |  | Salinity × Growth stage | 4 | 4.654 | 0.005 |

Table S2 Results of two‑way ANOVA for C, N stoichiometry in different plant parts

| Components | Growth part | Source of variation | DF | *F* | *P* |
| --- | --- | --- | --- | --- | --- |
| C | Aboveground | Salinity | 4 | 7.128 | 0.000 |
|  |  | Growth stage | 1 | 14.970 | 0.001 |
|  |  | Salinity × Growth stage | 4 | 3.024 | 0.034 |
|  | Belowground | Salinity | 4 | 0.143 | 0.964 |
|  |  | Growth stage | 1 | 0.629 | 0.435 |
|  |  | Salinity × Growth stage | 4 | 0.221 | 0.925 |
| N | Aboveground | Salinity | 4 | 4.761 | 0.005 |
|  |  | Growth stage | 1 | 52.382 | 0.000 |
|  |  | Salinity × Growth stage | 4 | 6.954 | 0.001 |
|  | Belowground | Salinity | 4 | 4.761 | 0.005 |
|  |  | Growth stage | 1 | 52.382 | 0.000 |
|  |  | Salinity × Growth stage | 4 | 6.954 | 0.001 |
| C:N | Aboveground | Salinity | 4 | 4.905 | 0.004 |
|  |  | Growth stage | 1 | 35.842 | 0.000 |
|  |  | Salinity × Growth stage | 4 | 6.500 | 0.001 |
|  | Belowground | Salinity | 4 | 2.569 | 0.059 |
|  |  | Growth stage | 1 | 48.382 | 0.000 |
|  |  | Salinity × Growth stage | 4 | 3.037 | 0.033 |

Table S3 The *P* values and relative contributions of Monte Carlo permutation tests for each soil factor

| Growth stage | Growth part | Indicator | SC | SN | SC:N | pH | SEC |
| --- | --- | --- | --- | --- | --- | --- | --- |
| The early growth stage | Aboveground | *P* | 0.352 | 0.498 | 0.144 | 0.952 | 0.190 |
|  |  | Contribution | 17.6% | 15.7% | 35.5% | 3.4% | 27.8% |
|  | Belowground | *P* | 0.140 | 0.836 | 0.050 | 0.958 | 0.620 |
|  |  | Contribution | 32.7% | 4.1% | 55.7% | 1.0% | 6.5% |
| The late growth stage | Aboveground | *P* | 0.280 | 0.904 | 0.630 | 0.072 | 0.064 |
|  |  | Contribution | 17.3% | 3.4% | 8.1% | 29.6% | 41.6% |
|  | Belowground | *P* | 0.148 | 0.874 | 0.104 | 0.062 | 0.916 |
|  |  | Contribution | 26.8% | 4.5% | 28.3% | 36.9% | 3.5% |

SC, soil C content; SN, soil N content; SC:N, soil C:N ratio; pH, soil pH; SEC, soil electrical conductivity.





Figure S1 Comparisons of soluble sugar, starch, and total NSC contents between growth stages. Different lowercase letters indicate significant differences in the same part.





Figure S2 Comparisons of C, N stoichiometry between growth stages. Different lowercase letters indicate significant differences in the same part.
